# Effect of denosumab, a human monoclonal antibody of receptor activator of nuclear factor kappa-B ligand (RANKL), upon glycemic and metabolic parameters: Effect of denosumab on glycemic parameters

**DOI:** 10.1097/MD.0000000000018067

**Published:** 2019-11-22

**Authors:** Ichiro Abe, Kentaro Ochi, Yuichi Takashi, Yuka Yamao, Hanako Ohishi, Hideyuki Fujii, Midori Minezaki, Kaoru Sugimoto, Tadachika Kudo, Makiko Abe, Yasushi Ohnishi, Shigeaki Mukoubara, Kunihisa Kobayashi

**Affiliations:** a Department of Endocrinology and Diabetes Mellitus, Fukuoka University Chikushi Hospital, Chikushino, Fukuoka; b Department of Internal Medicine, Nagasaki Prefecture Iki Hospital, Iki, Nagasaki; c Department of Preventive Medicine, Kyushu University Faculty of Medical Sciences, Fukuoka, Japan.

**Keywords:** denosumab receptor activator of nuclear factor kappa-B ligand, osteoporosis, type 2 diabetes, insulin resistance

## Abstract

Osteoporosis is a complication of type 2 diabetes mellitus (T2DM). Blockade of receptor activator of nuclear factor kappa-B ligand (RANKL) improves osteoporosis, but might also improve glucose tolerance through reduction of hepatic insulin resistance. However, the effect of denosumab (a human monoclonal antibody of RANKL) upon glycemic and metabolic parameters is controversial. We revealed the effect of denosumab upon glycemic and metabolic parameters for 52 weeks. We evaluated 20 individuals diagnosed with both osteoporosis (male and female: postmenopausal) and T2DM. We measured glycemic and metabolic parameters before and 26/52 weeks after administration of denosumab (60 mg per 26 weeks) without changing any other medication each patient was taking. All patients completed the study without complications and the T-score (lumbar spine and femoral neck) improved significantly from baseline to 52 weeks after denosumab administration (*P* < .001, .001, respectively). None of the glycemic parameters changed significantly from baseline to 26 weeks after denosumab administration, but levels of glycated hemoglobin and homeostasis model assessment of insulin resistance improved significantly from baseline to 52 weeks after administration (*P* = .019, .008, respectively). The levels of liver enzymes did not change significantly from baseline to 26 weeks after denosumab administration, but levels of aspartate transaminase and alanine aminotransferase improved significantly from baseline to 52 weeks after administration (*P* = .014, .004, respectively). None of the markers of lipid metabolism and body mass index changed significantly from baseline to 26/52 weeks after denosumab administration. These data demonstrated that denosumab is useful for T2DM patients with osteoporosis for glycemic control *via* improvement of insulin resistance. Also, the effect of denosumab might be due to improvement of hepatic function.

## 1 Introduction

Type 2 diabetes mellitus (T2DM) is a chronic metabolic disease. The increasing prevalence of T2DM worldwide is considered to be an important healthcare issue.^[1–3]^

Osteoporosis is a complication of T2DM.^[4,5]^ Osteoporosis is characterized by a decrease in bone mass and disruption of bone architecture, resulting in an increased risk of fragility fractures.^[6]^ T2DM affects the metabolism and strength of bone directly.^[4]^ Accordingly, good control of T2DM is important to manage osteoporosis and vice versa.

Just like the development of medications for T2DM, medications for osteoporosis have been developed recently.^[7]^ Denosumab is used to treat osteoporosis. Denosumab is a human monoclonal antibody that binds to the receptor activator of nuclear factor kappa-B ligand (RANKL). RANKL plays an important part in mediating bone resorption by accelerating the formation and function of osteoclasts.^[8]^ The efficacy of denosumab against osteoporosis was revealed by a large randomized clinical trial of long duration: Fracture Reduction Evaluation of Denosumab in Osteoporosis every 6 Months (FREEDOM).^[9]^

Kiechi and co-authors reported that blockade of RANKL improved hepatic insulin resistance and prevented DM development in an in vivo study.^[10]^ However, the effect of denosumab upon glycemic and metabolic parameters in humans is not clear. Two studies of short duration (14 patients for 12 weeks, and 48 patients for 24 weeks) showed denosumab did not improve glycemic parameters sufficiently,^[11,12]^ and 1 study of long duration (*post hoc* analysis of the FREEDOM trial) showed denosumab improved fasting serum glucose levels only in patients with T2DM who were not on anti-DM agents (that study investigated only the fasting serum glucose level as a glycemic parameter).^[13]^

In the present study, we investigated the effect of denosumab upon glycemic and metabolic parameters of patients with T2DM for 52 weeks.

## 2 Materials and methods

### 2.1 Ethical approval of the study protocol

All participants provided written informed consent for study inclusion. The study protocol was approved by the Ethics Review Committee of Nagasaki Prefecture Iki Hospital (Nagasaki, Japan).

### 2.2 Study participants

We recruited 20 individuals diagnosed with osteoporosis (male and female: postmenopausal) and T2DM at Nagasaki Prefecture Iki Hospital from July 2013 to August 2018. The diagnosis of osteoporosis was made in accordance with criteria used widely, as described previously.^[14]^ DM was defined as any combination of fasting plasma glucose ≥126 mg/dl, random plasma glucose ≥200 mg/dl, glycated hemoglobin (HbA_1c_) ≥6.5%, or use of anti-DM agents. Participant characteristics are shown in Table 1. Exclusion criteria were patients who were (or might have been) pregnant, have (or had) cancer, or were receiving insulin therapy.

**Table 1:**
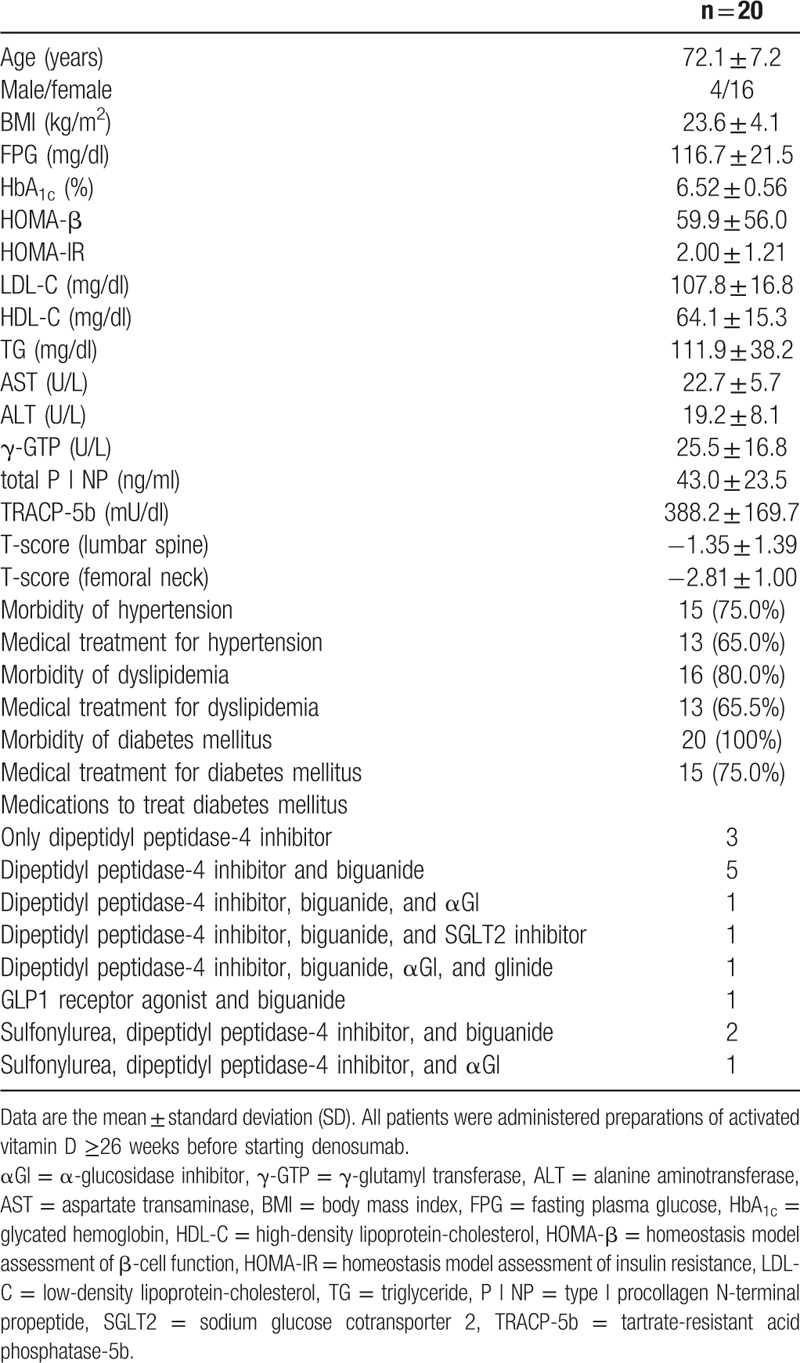
Clinical characteristics of the patient cohort.

### 2.3 Methods

To examine the effect of denosumab (60 mg per 26 weeks), we administered and continued treatment for 52 weeks. The following variables were measured at baseline, 26 weeks after, and 52 weeks after administration of denosumab: parameters of glucose control (HbA_1c_, fasting plasma glucose (FPG), homeostasis model assessment of insulin resistance (HOMA-IR), homeostasis model assessment of β-cell function (HOMA-β); markers of lipid metabolism (low-density lipoprotein-cholesterol (LDL-C), high-density lipoprotein cholesterol (HDL-C), and triglycerides (TG)); liver enzymes (aspartate transaminase (AST), alanine transaminase (ALT), gamma-glutamyl transferase (γ-GTP)) and body mass index (BMI). Blood samples were obtained after an overnight fast, and HOMA-IR was calculated using the following formula: 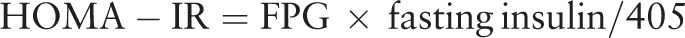


HOMA-β was calculated using the following formula:
 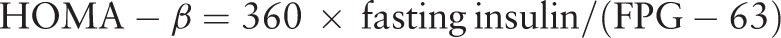


It is common to administer dietary/activated vitamin D together to prevent the hypocalcemia caused by denosumab.^[15]^ However, vitamin D could affect glucose tolerance,^[16–18]^ so our patients were started on activated vitamin D ≥26 weeks before the first administration of denosumab and continued taking it during the study. In addition, the other drugs being taken for osteoporosis were stopped ≥26 weeks before the first administration of denosumab. None of the other drugs being taken (except those being taken to treat osteoporosis) were changed during our study. With regard to the effect on osteoporosis by denosumab, values of the T-score (lumbar spine and femoral neck) were measured at baseline and 52 weeks after denosumab administration.

### 2.4 Statistical analyses

Data are the mean ± standard deviation (SD). The significance of differences between mean values was estimated by paired *t*-test with Dunnett correction. *P* < .05 (for the T-score)/*P* < .025 (for parameters except the T-score) was considered significant.

## 3 Results

All patients completed the present study without complications. None of the glycemic parameters changed significantly from baseline to 26 weeks after denosumab administration (FPG: 116.7 ± 21.5 vs 119.0 ± 22.0 mg/dl, *P* = .273; HbA_1c_: 6.52 ± 0.56% vs 6.48 ± 0.60%, *P* = .284; HOMA-β: 59.9 ± 56.0 vs 41.8 ± 25.3, *P* = .051; HOMA-IR: 2.00 ± 1.21 vs 1.66 ± 0.98, *P* = .066, respectively). However, levels of HbA_1c_ and HOMA-IR improved significantly from baseline to 52 weeks after denosumab administration (HbA_1c_: 6.52 ± 0.56% *vs*. 6.32 ± 0.61%, *P* = .019; HOMA-IR: 2.00 ± 1.21 vs 1.38 ± 0.76, *P* = .008). FPG improved from baseline to 52 weeks after denosumab administration but not significantly (116.7 ± 21.5 vs 109.3 ± 18.5 mg/dl, *P* = .048). With regard to HOMA-β, there was no significant difference between values at baseline and 52 weeks after denosumab administration (HOMA-β: 59.9 ± 56.0 vs 44.2 ± 25.1, *P* = .070).

None of the markers of lipid metabolism changed significantly from baseline to 26/52 weeks after administration of denosumab (LDL-C: 107.8 ± 16.8 vs 108.8 ± 20.1/105.5 ± 20.8 mg/dl, *P* = .391/.204; HDL-C: 64.1 ± 15.3 vs 66.8 ± 17.4/65.1 ± 15.2 mg/dl, *P* = .069/.314; TG; 111.9 ± 38.2 vs 113.8 ± 46.7/108.1 ± 40.2 mg/dl, *P* = .422/.231, respectively).

Levels of liver enzymes did not change significantly from baseline to 26 weeks after administration of denosumab (AST: 22.7 ± 5.7 vs 22.9 ± 5.6 U/L, *P* = .404; ALT: 19.2 ± 8.1 vs 18.4 ± 7.4 U/L, *P* = .190; γ-GTP: 25.5 ± 16.8 vs 26.7 ± 16.0 U/L, *P* = .279, respectively). However, levels of AST and ALT improved significantly from baseline to 52 weeks after denosumab administration (AST: 22.7 ± 5.7 vs 20.9 ± 4.8 U/L, *P* = .014; ALT: 19.2 ± 8.1 vs 16.6 ± 6.6 U/L, *P* = .004) whereas γ-GTP levels did not change significantly (γ-GTP: 25.5 ± 16.8 *vs*. 23.7 ± 14.5 U/L, *P* = .123).

There was no significant difference in BMI from baseline and 26/52 weeks after administration of denosumab (23.6 ± 4.1 vs 22.7 ± 5.6/23.4 ± 4.1 kg/m^2^, *P* = .198/.161, respectively).

In terms of osteoporosis, the T-score of the lumbar spine and femoral neck improved significantly from baseline to 52 weeks after administration of denosumab (lumbar spine: − 1.35 ± 1.39 vs − 0.91 ± 1.55, *P* < .001; femoral neck: − 2.81 ± 1.00 vs − 2.45 ± 1.02, *P* = .001). All changes in parameters are shown in Table 2.

**Table 2:**
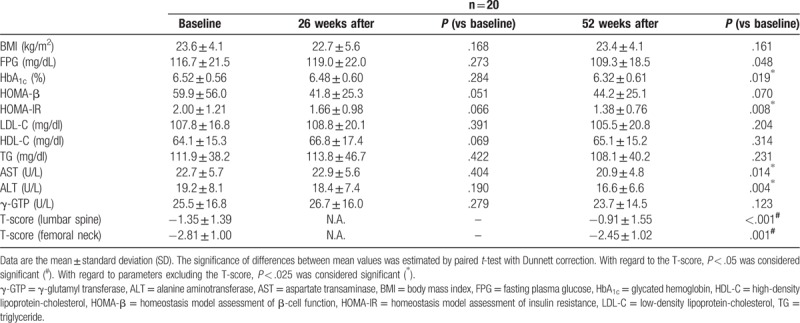
Glycemic parameters, metabolic parameters, and T-scores at baseline and 26/52 weeks after denosumab administration in the study cohort.

## 4 Discussion

With the increasing prevalence of T2DM comes an increase in the importance of preventing its complications.^[1–3]^ Osteoporosis is one of the complications of T2DM,^[4,5]^ and it is necessary to treat osteoporosis to prevent fragility fractures.

Several types of drugs can be used to treat osteoporosis, including denosumab. Denosumab has been reported to inhibit bone resorption, and its long-term efficacy has been reported.^[9,19]^ Conversely, denosumab might improve glucose tolerance through reduction of hepatic insulin resistance. Kiechl and co-workers reported that blockade of RANKL can improve hepatic insulin resistance and prevent DM development in an in vivo study.^[10]^

In humans, the effects of denosumab on glycemic and metabolic parameters is controversial. Two studies of short duration did not reveal sufficient improvement of glycemic parameters by denosumab.^[11,12]^*Post hoc* analysis of the FREEDOM study did not reveal improvement of glycemic parameters by denosumab initially,^[20]^ but further analysis by Napoli and colleagues demonstrated that denosumab improved fasting serum glucose levels only in patients with T2DM who were not taking anti-DM agents.^[13]^ Their study was a long-term observation but investigated the fasting serum glucose level only.

We investigated the effects of denosumab upon the glycemic and metabolic parameters of patients with T2DM for 52 weeks. At first, our results showed no changes in glycemic or metabolic parameters between baseline and 26 weeks after administration of denosumab. These data were almost identical to those in 2 studies of short duration. However, our study showed levels of HbA_1c_ and HOMA-IR to be improved significantly from baseline to 52 weeks after administration of denosumab. Furthermore, levels of AST and ALT improved significantly from baseline to 52 weeks after administration of denosumab. Considering these data and no change in HOMA-β, improvement of glycemic control might be due to improvement of insulin resistance. In addition, improvement of AST/ALT levels and no changes in BMI suggests that the effect of denosumab on insulin resistance might be caused by improvement of hepatic insulin resistance, similar to the results documented in the in vivo study by Kiechl and colleagues.^[10]^ Accordingly, our study might reveal the effect of denosumab upon the glycemic and metabolic parameters of patients with T2DM, including its mechanism of action. With regard to FPG, there were no significant changes in our study. Meanwhile, significant improvement of levels of HbA_1c_ without significant improvement of FPG might indicate improvement of postprandial hyperglycemia. Amelioration of insulin resistance was reported to lead to improvement of postprandial hyperglycemia compared with that of impaired fasting glycemia,^[21]^ and longer-term studies can reveal significant changes in FPG as well as the previous analysis of the FREEDOM trial.^[13]^

Our study protocol was different to that of other reports. Patients in our study started taking activated vitamin D ≥ 26 weeks before the first administration of denosumab and continued during the study. Commonly, to prevent the hypocalcemia caused by denosumab, patients are administered dietary/activated vitamin D together.^[15]^ However, it has been reported that administration of vitamin D might improve glucose tolerance in humans.^[18]^ Furthermore, it has been reported that administration of vitamin D might improve fatty liver disease and hepatic insulin resistance.^[22]^ While other studies have not considered this factor, additional effect of vitamin D to results could be excluded in our study. Thus, our study could reveal efficacy on glycemic parameters and hepatic function properly.

Our study had 2 main limitations. First, our study cohort was small because we recruited patients with both T2DM and osteoporosis who were not having insulin therapy so that we could employ HOMA-IR and HOMA-β as surrogate markers of insulin resistance and insulin secretion. Second, we employed HOMA-IR and HOMA-β as surrogate markers of insulin resistance and insulin secretion as a substitute for hyperglycemic/hyperinsulinemic–euglycemic clamps. Thus, future studies, particularly investigations of large numbers and for longer period, are required to confirm our results.

## 5 Conclusions

We revealed that denosumab is useful for patients with both T2DM and osteoporosis for glycemic control possibly due to improvement of insulin resistance, possibly by improvement of hepatic function, as well as osteoporosis itself.

## Acknowledgments

We thank Ms. Yumi Iriguchi, Ms. Akiko Harada, Mr. Nobuhiro Tashima, and Mr. Yasuyuki Matsumoto for assistance in undertaking our study.

## Author contributions

**Conceptualization:** Ichiro Abe, Kentaro Ochi, Yasushi Ohnishi, Shigeaki Mukoubara, Kunihisa Kobayashi.

**Data curation:** Ichiro Abe, Kentaro Ochi.

**Formal analysis:** Ichiro Abe, Kentaro Ochi, Makiko Abe, Kunihisa Kobayashi.

**Investigation:** Ichiro Abe, Kentaro Ochi.

**Methodology:** Ichiro Abe.

**Project administration:** Ichiro Abe, Yasushi Ohnishi, Shigeaki Mukoubara.

**Supervision:** Yuichi Takashi, Yuka Yamao, Hanako Ohishi, Hideyuki Fujii, Midori Minezaki, Kaoru Sugimoto, Tadachika Kudo, Makiko Abe, Yasushi Ohnishi, Shigeaki Mukoubara, Kunihisa Kobayashi.

**Validation:** Ichiro Abe, Kentaro Ochi, Yuichi Takashi, Yuka Yamao, Hanako Ohishi, Hideyuki Fujii, Midori Minezaki, Kaoru Sugimoto, Tadachika Kudo, Makiko Abe, Yasushi Ohnishi, Shigeaki Mukoubara, Kunihisa Kobayashi.

**Visualization:** Ichiro Abe, Kentaro Ochi, Yuichi Takashi, Yuka Yamao, Hanako Ohishi, Hideyuki Fujii, Midori Minezaki, Kaoru Sugimoto, Tadachika Kudo, Makiko Abe, Yasushi Ohnishi, Shigeaki Mukoubara, Kunihisa Kobayashi.

**Writing – original draft:** Ichiro Abe.

**Writing – review & editing:** Ichiro Abe, Kentaro Ochi, Yuichi Takashi, Yuka Yamao, Hanako Ohishi, Hideyuki Fujii, Midori Minezaki, Kaoru Sugimoto, Tadachika Kudo, Makiko Abe, Yasushi Ohnishi, Shigeaki Mukoubara, Kunihisa Kobayashi.

Ichiro Abe orcid: 0000-0002-7545-9751.
